# Di-*n*-butyl­ammonium 2-[(3,5-di-*tert*-butyl-4-hy­droxy­benz­yl)sulfan­yl]benzoate

**DOI:** 10.1107/S1600536810033921

**Published:** 2010-08-28

**Authors:** Abeer A. Alhadi, Hamid Khaledi, Hapipah Mohd Ali, Marilyn M. Olmstead

**Affiliations:** a Department of Chemistry, University of Malaya, 50603 Kuala Lumpur, Malaysia; b Department of Chemistry, University of California, One Shields Avenue, Davis, CA 95616, USA

## Abstract

The title salt, C_8_H_20_N^+^·C_22_H_27_O_3_S^−^, is a proton-transfer compound derived from the recently reported parent carb­oxy­lic acid [Alhadi *et al. *(2010). *Acta Cryst.* E**66**, o1787] by the addition of a second equivalent of di-*n*-butyl­amine, yielding the di-*n*-butyl­ammonium carboxyl­ate salt. The structure of the carboxyl­ate anion resembles that of the parent carb­oxy­lic acid. The main difference lies in the position of the H atom in the 4-hy­droxy group. In the anion the O—H bond is perpendicular, rather than parallel, to the benzyl ring. This position appears to facilitate hydrogen bonding to an O atom of the carboxyl­ate group of a symmetry-related anion. In addition, there are three N—H⋯O hydrogen bonds. In contrast, the neutral species hydrogen bonds *via* a carboxylic acid dimer. The dihedral angle between the benzene rings in the anion is 79.19 (7)°.

## Related literature

For the structure of the parent benzoic acid, see: Alhadi *et al.* (2010 ▶). For a similar structure based on nicotinic acid, see: Mansor *et al.* (2008 ▶).
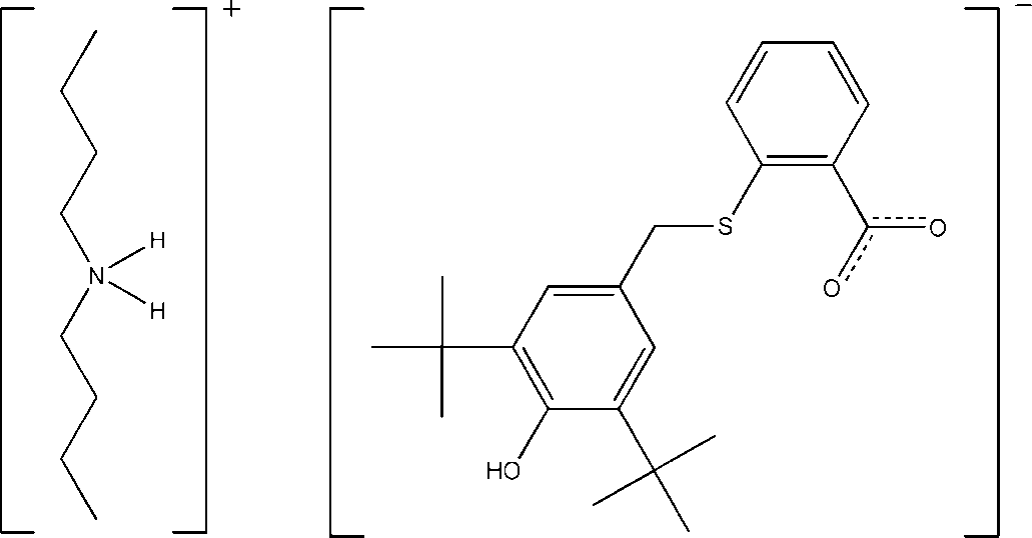


## Experimental

### 

#### Crystal data

C_8_H_20_N^+^·C_22_H_27_O_3_S^−^
*M* *_r_* = 501.75
Orthorhombic, 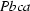

*a* = 12.8631 (5) Å
*b* = 20.1109 (9) Å
*c* = 23.0930 (9) Å
*V* = 5973.9 (4) Å^3^
*Z* = 8
Mo *K*α radiation
μ = 0.14 mm^−1^
*T* = 296 K
0.60 × 0.40 × 0.35 mm

#### Data collection

Bruker APEXII diffractometer
Absorption correction: multi-scan (*SADABS*; Sheldrick, 1996 ▶) *T*_min_ = 0.922, *T*_max_ = 0.954
44646 measured reflections
5277 independent reflections
3311 reflections with *I* > 2σ(*I*)
*R*_int_ = 0.080

#### Refinement

*R*[*F* ^2^ > 2σ(*F* ^2^)] = 0.050
*wR*(*F* ^2^) = 0.138
*S* = 1.01
5277 reflections
333 parameters
3 restraints
H atoms treated by a mixture of independent and constrained refinement
Δρ_max_ = 0.28 e Å^−3^
Δρ_min_ = −0.17 e Å^−3^

### 

Data collection: *APEX2* (Bruker, 2007 ▶); cell refinement: *SAINT* (Bruker, 2007 ▶); data reduction: *SAINT*; program(s) used to solve structure: *SHELXS97* (Sheldrick, 2008 ▶); program(s) used to refine structure: *SHELXL97* (Sheldrick, 2008 ▶); molecular graphics: *XP* (Bruker, 2007 ▶); software used to prepare material for publication: *publCIF* (Westrip, 2010 ▶).

## Supplementary Material

Crystal structure: contains datablocks I, global. DOI: 10.1107/S1600536810033921/bh2302sup1.cif

Structure factors: contains datablocks I. DOI: 10.1107/S1600536810033921/bh2302Isup2.hkl

Additional supplementary materials:  crystallographic information; 3D view; checkCIF report

## Figures and Tables

**Table 1:** Hydrogen-bond geometry (Å, °)

*D*—H⋯*A*	*D*—H	H⋯*A*	*D*⋯*A*	*D*—H⋯*A*
O3—H3⋯O1^i^	0.81 (2)	2.02 (2)	2.723 (3)	146 (3)
N1—H1*A*⋯O2	0.89 (2)	1.88 (2)	2.767 (3)	177 (3)
N1—H1*B*⋯O1^ii^	0.89 (2)	2.00 (2)	2.806 (3)	150 (3)
N1—H1*B*⋯O2^ii^	0.89 (2)	2.36 (2)	3.167 (3)	150 (2)
N1—H1*B*⋯O2^ii^	0.89 (2)	2.36 (2)	3.167 (3)	150 (2)

Symmetry codes: (i) 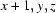; (ii) 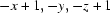.

**Fig. 1.**
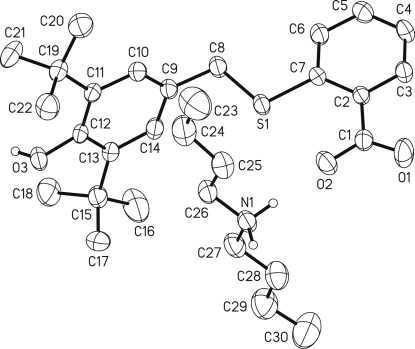
Thermal ellipsoid plot of the title compound at the 30% probability level. H atoms participating in hydrogen bonding are drawn as spheres of arbitrary radius.

**Fig. 2.**
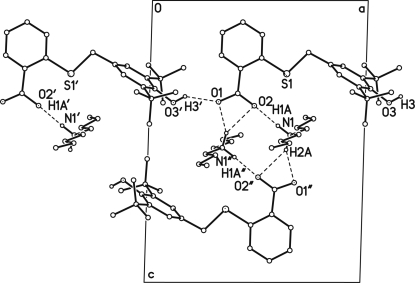
A view of the hydrogen bonding interactions (dashed lines) as viewed down b. Symmetry codes: ' = x - 1, y, z; " = -x + 1, -y, -z + 1.

## supplementary crystallographic information

### Comment

As shown in Fig. 1, the title compound is a di-*n*-butylammonium salt. It is a proton transfer derivative of the previously reported benzoic acid analog (Alhadi *et al.*, 2010). In contrast to the structure of the parent carboxylic acid in which the C—O—H fragment was found coplanar to the aromatic ring and therefore not involved in any hydrogen bonding, in the present structure the O—H bond is perpendicular to the aromatic ring, and it participates in hydrogen bonding to the carboxylate group of the neighboring anion, forming an infinite chain along the *a* axis (Fig. 2). This arrangement is similar to that reported for the salt based on nicotinic acid (Mansor *et al.*, 2008). The dihedral angle between the aromatic rings C2/C3/C4/C5/C6/C7 and C9/C10/C11/C12/C13/C14 is 79.19 (7)°.

### Experimental

Thiosalicylic acid (0.154 g, 1 mmol), 2,6-di-*t*-butylphenol (2.00 g, 1 mmol) and *para*formaldehyde (0.291 g, 1 mmol) were ground into a homogenous powder and to this was added di-*n*-butylamine (0.1 ml). The slurry was heated to 353 K for 2.5 h, then cooled to 323 K. Ethanol (20 ml) was added and the mixture was stirred for 1 h at room temperature. To the resulting clear solution di-*n*-butylamine (0.1 ml) was added and the solution was set aside in the dark for 5 days whereupon the colorless crystals of the title compound were obtained.

### Refinement

The C-bound hydrogen atoms were placed at calculated positions (C—H 0.93–0.97 Å) and were treated as riding on their parent atoms. The nitrogen- and oxygen-bound hydrogen atoms were located in a difference map and were refined freely with distances restrained to N—H 0.86 (2) and O—H 0.82 (2) Å. For all H atoms, *U*_iso_(H) was set to 1.2–1.5 *U*_eq_(carrier atom).

### Crystal data

**Table:**

C_8_H_20_N^+^·C_22_H_27_O_3_S^−^	*F*(000) = 2192
*M**_r_* = 501.75	*D*_x_ = 1.116 Mg m^−^^3^
Orthorhombic, *P**b**c**a*	Mo *K*α radiation, λ = 0.71073 Å
Hall symbol: -P 2ac 2ab	Cell parameters from 3944 reflections
*a* = 12.8631 (5) Å	θ = 2.4–19.4°
*b* = 20.1109 (9) Å	µ = 0.14 mm^−^^1^
*c* = 23.0930 (9) Å	*T* = 296 K
*V* = 5973.9 (4) Å^3^	Irregular block, colourless
*Z* = 8	0.60 × 0.40 × 0.35 mm

### Data collection

**Table:**

Bruker APEXII diffractometer	5277 independent reflections
Radiation source: fine-focus sealed tube	3311 reflections with *I* > 2σ(*I*)
graphite	*R*_int_ = 0.080
φ and ω scans	θ_max_ = 25.0°, θ_min_ = 1.8°
Absorption correction: multi-scan (*SADABS*; Sheldrick, 1996)	*h* = −15→15
*T*_min_ = 0.922, *T*_max_ = 0.954	*k* = −23→23
44646 measured reflections	*l* = −27→27

### Refinement

**Table:**

Refinement on *F*^2^	Primary atom site location: structure-invariant direct methods
Least-squares matrix: full	Secondary atom site location: difference Fourier map
*R*[*F*^2^ > 2σ(*F*^2^)] = 0.050	Hydrogen site location: inferred from neighbouring sites
*wR*(*F*^2^) = 0.138	H atoms treated by a mixture of independent and constrained refinement
*S* = 1.01	*w* = 1/[σ^2^(*F*_o_^2^) + (0.0533*P*)^2^ + 2.2704*P*] where *P* = (*F*_o_^2^ + 2*F*_c_^2^)/3
5277 reflections	(Δ/σ)_max_ < 0.001
333 parameters	Δρ_max_ = 0.28 e Å^−^^3^
3 restraints	Δρ_min_ = −0.17 e Å^−^^3^
0 constraints	

### Fractional atomic coordinates and isotropic or equivalent isotropic displacement parameters (Å^2^)

**Table:**

	*x*	*y*	*z*	*U*_iso_*/*U*_eq_	
C1	0.4142 (2)	0.10279 (13)	0.41431 (11)	0.0573 (7)	
S1	0.64187 (5)	0.14037 (4)	0.37500 (3)	0.0571 (2)	
O1	0.32489 (16)	0.08674 (12)	0.42807 (11)	0.1060 (9)	
O2	0.49152 (15)	0.07570 (10)	0.43550 (9)	0.0799 (6)	
O3	1.11548 (13)	0.06947 (10)	0.41947 (9)	0.0676 (5)	
H3	1.1701 (18)	0.0878 (16)	0.4133 (14)	0.101*	
N1	0.65789 (17)	0.01880 (12)	0.49304 (11)	0.0639 (6)	
H1A	0.6030 (17)	0.0360 (13)	0.4749 (11)	0.077*	
H1B	0.639 (2)	−0.0157 (11)	0.5148 (11)	0.077*	
C2	0.42822 (17)	0.15713 (11)	0.37050 (9)	0.0443 (6)	
C3	0.34012 (19)	0.18891 (13)	0.34973 (11)	0.0552 (6)	
H3A	0.2754	0.1757	0.3635	0.066*	
C4	0.3448 (2)	0.23882 (15)	0.30980 (12)	0.0669 (8)	
H4	0.2843	0.2591	0.2966	0.080*	
C5	0.4395 (2)	0.25874 (15)	0.28939 (13)	0.0732 (9)	
H5	0.4436	0.2928	0.2623	0.088*	
C6	0.5294 (2)	0.22837 (14)	0.30893 (12)	0.0648 (7)	
H6	0.5934	0.2422	0.2945	0.078*	
C7	0.52605 (17)	0.17767 (12)	0.34970 (10)	0.0459 (6)	
C8	0.74329 (17)	0.18495 (13)	0.33648 (11)	0.0579 (7)	
H8A	0.7354	0.1789	0.2950	0.069*	
H8B	0.7394	0.2321	0.3450	0.069*	
C9	0.84580 (18)	0.15715 (13)	0.35632 (11)	0.0512 (6)	
C10	0.89894 (18)	0.11082 (12)	0.32410 (11)	0.0516 (6)	
H10	0.8716	0.0979	0.2885	0.062*	
C11	0.99201 (17)	0.08236 (12)	0.34249 (10)	0.0482 (6)	
C12	1.02979 (17)	0.10199 (12)	0.39672 (10)	0.0486 (6)	
C13	0.97987 (18)	0.15064 (13)	0.43035 (10)	0.0524 (6)	
C14	0.88760 (18)	0.17683 (13)	0.40858 (11)	0.0556 (7)	
H14	0.8527	0.2089	0.4302	0.067*	
C15	1.0201 (2)	0.17282 (17)	0.49001 (12)	0.0734 (9)	
C16	0.9545 (3)	0.2305 (2)	0.51411 (17)	0.1397 (19)	
H16A	0.9577	0.2675	0.4879	0.209*	
H16B	0.9814	0.2436	0.5512	0.209*	
H16C	0.8837	0.2164	0.5183	0.209*	
C17	1.0103 (3)	0.1149 (2)	0.53240 (13)	0.1155 (15)	
H17A	1.0507	0.0780	0.5186	0.173*	
H17B	0.9387	0.1020	0.5355	0.173*	
H17C	1.0354	0.1284	0.5697	0.173*	
C18	1.1328 (2)	0.19722 (19)	0.48760 (15)	0.0960 (11)	
H18A	1.1779	0.1605	0.4790	0.144*	
H18B	1.1516	0.2161	0.5243	0.144*	
H18C	1.1395	0.2304	0.4579	0.144*	
C19	1.04977 (19)	0.03203 (13)	0.30369 (11)	0.0579 (7)	
C20	0.9917 (2)	0.02028 (16)	0.24637 (13)	0.0822 (9)	
H20A	1.0298	−0.0109	0.2231	0.123*	
H20B	0.9854	0.0616	0.2258	0.123*	
H20C	0.9237	0.0028	0.2543	0.123*	
C21	1.1572 (2)	0.05973 (17)	0.28782 (13)	0.0846 (10)	
H21A	1.2001	0.0610	0.3218	0.127*	
H21B	1.1497	0.1039	0.2725	0.127*	
H21C	1.1889	0.0317	0.2592	0.127*	
C22	1.0605 (2)	−0.03551 (15)	0.33351 (14)	0.0824 (9)	
H22A	1.0975	−0.0655	0.3086	0.124*	
H22B	0.9926	−0.0532	0.3415	0.124*	
H22C	1.0980	−0.0302	0.3691	0.124*	
C23	0.7166 (4)	−0.1192 (2)	0.3170 (2)	0.1382 (17)	
H23A	0.6944	−0.1593	0.3359	0.207*	
H23B	0.7700	−0.1295	0.2894	0.207*	
H23C	0.6585	−0.0993	0.2974	0.207*	
C24	0.7569 (3)	−0.0731 (3)	0.35968 (18)	0.1261 (15)	
H24A	0.7790	−0.0331	0.3397	0.151*	
H24B	0.8183	−0.0928	0.3771	0.151*	
C25	0.6833 (3)	−0.05329 (18)	0.40777 (15)	0.0897 (10)	
H25A	0.6222	−0.0325	0.3910	0.108*	
H25B	0.6608	−0.0929	0.4283	0.108*	
C26	0.7327 (2)	−0.00614 (17)	0.44985 (13)	0.0771 (9)	
H26A	0.7892	−0.0286	0.4696	0.093*	
H26B	0.7619	0.0312	0.4288	0.093*	
C27	0.7013 (2)	0.06935 (16)	0.53258 (14)	0.0783 (9)	
H27A	0.7257	0.1071	0.5102	0.094*	
H27B	0.7603	0.0507	0.5530	0.094*	
C28	0.6206 (3)	0.09282 (17)	0.57620 (15)	0.0901 (10)	
H28A	0.5647	0.1150	0.5557	0.108*	
H28B	0.5914	0.0544	0.5957	0.108*	
C29	0.6642 (3)	0.1391 (2)	0.62032 (18)	0.1121 (13)	
H29A	0.6988	0.1755	0.6006	0.135*	
H29B	0.7160	0.1157	0.6430	0.135*	
C30	0.5837 (3)	0.1671 (3)	0.6604 (2)	0.1449 (18)	
H30A	0.5335	0.1918	0.6384	0.217*	
H30B	0.6164	0.1959	0.6880	0.217*	
H30C	0.5494	0.1314	0.6804	0.217*	

### Atomic displacement parameters (Å^2^)

**Table:**

	*U*^11^	*U*^22^	*U*^33^	*U*^12^	*U*^13^	*U*^23^
C1	0.0437 (15)	0.0638 (17)	0.0644 (17)	0.0003 (14)	0.0004 (13)	0.0071 (14)
S1	0.0381 (3)	0.0675 (4)	0.0658 (4)	0.0052 (3)	−0.0047 (3)	0.0172 (3)
O1	0.0492 (12)	0.1187 (19)	0.150 (2)	−0.0007 (12)	0.0137 (13)	0.0679 (17)
O2	0.0560 (12)	0.0903 (15)	0.0934 (15)	0.0010 (11)	−0.0136 (10)	0.0372 (12)
O3	0.0384 (10)	0.0833 (14)	0.0810 (13)	0.0067 (10)	−0.0051 (9)	0.0132 (11)
N1	0.0461 (13)	0.0715 (17)	0.0741 (17)	−0.0010 (12)	−0.0089 (12)	0.0213 (13)
C2	0.0394 (13)	0.0497 (14)	0.0437 (13)	0.0041 (11)	−0.0048 (10)	−0.0035 (11)
C3	0.0408 (14)	0.0645 (17)	0.0603 (16)	0.0078 (12)	−0.0032 (12)	−0.0009 (14)
C4	0.0497 (16)	0.080 (2)	0.0707 (18)	0.0224 (15)	−0.0118 (13)	0.0079 (16)
C5	0.0632 (18)	0.080 (2)	0.076 (2)	0.0161 (16)	−0.0034 (15)	0.0288 (16)
C6	0.0465 (15)	0.0763 (19)	0.0715 (18)	0.0088 (14)	0.0009 (13)	0.0221 (16)
C7	0.0400 (13)	0.0513 (14)	0.0462 (14)	0.0064 (11)	−0.0047 (10)	0.0040 (12)
C8	0.0407 (14)	0.0651 (17)	0.0679 (17)	0.0015 (13)	−0.0041 (12)	0.0126 (14)
C9	0.0388 (13)	0.0586 (16)	0.0562 (15)	−0.0020 (12)	−0.0018 (11)	0.0095 (12)
C10	0.0429 (13)	0.0609 (16)	0.0510 (15)	−0.0045 (13)	−0.0015 (11)	0.0036 (13)
C11	0.0379 (13)	0.0557 (15)	0.0512 (15)	−0.0006 (11)	0.0068 (11)	0.0065 (12)
C12	0.0309 (12)	0.0614 (16)	0.0536 (15)	−0.0005 (12)	0.0056 (11)	0.0088 (12)
C13	0.0375 (13)	0.0700 (17)	0.0497 (15)	−0.0021 (12)	0.0020 (11)	0.0026 (13)
C14	0.0413 (14)	0.0644 (17)	0.0612 (17)	0.0033 (12)	0.0051 (12)	−0.0020 (13)
C15	0.0501 (16)	0.113 (2)	0.0569 (18)	0.0101 (17)	−0.0042 (13)	−0.0163 (17)
C16	0.106 (3)	0.208 (5)	0.105 (3)	0.055 (3)	−0.034 (2)	−0.092 (3)
C17	0.072 (2)	0.217 (5)	0.058 (2)	−0.007 (3)	−0.0067 (17)	0.031 (3)
C18	0.070 (2)	0.128 (3)	0.090 (2)	−0.016 (2)	−0.0125 (17)	−0.031 (2)
C19	0.0473 (15)	0.0646 (17)	0.0616 (17)	0.0025 (13)	0.0091 (12)	−0.0025 (14)
C20	0.084 (2)	0.091 (2)	0.071 (2)	0.0077 (18)	0.0033 (17)	−0.0192 (18)
C21	0.0661 (19)	0.102 (3)	0.086 (2)	−0.0117 (18)	0.0308 (16)	−0.0162 (19)
C22	0.079 (2)	0.069 (2)	0.099 (2)	0.0095 (17)	0.0052 (18)	0.0039 (18)
C23	0.154 (4)	0.123 (3)	0.138 (4)	0.040 (3)	−0.021 (3)	−0.026 (3)
C24	0.081 (3)	0.181 (5)	0.116 (3)	0.021 (3)	−0.007 (2)	−0.011 (3)
C25	0.071 (2)	0.099 (3)	0.099 (3)	0.0172 (19)	−0.0035 (19)	0.003 (2)
C26	0.0496 (17)	0.098 (2)	0.084 (2)	0.0130 (17)	−0.0005 (15)	0.0249 (19)
C27	0.0577 (18)	0.088 (2)	0.089 (2)	−0.0107 (17)	−0.0169 (16)	0.0154 (18)
C28	0.069 (2)	0.095 (3)	0.107 (3)	−0.0073 (19)	−0.0078 (19)	−0.009 (2)
C29	0.090 (3)	0.130 (3)	0.117 (3)	−0.010 (2)	−0.009 (2)	−0.021 (3)
C30	0.105 (3)	0.183 (5)	0.147 (4)	−0.021 (3)	0.022 (3)	−0.055 (4)

### Geometric parameters (Å, °)

**Table:**

C1—O2	1.235 (3)	C17—H17B	0.9600
C1—O1	1.235 (3)	C17—H17C	0.9600
C1—C2	1.500 (3)	C18—H18A	0.9600
S1—C7	1.767 (2)	C18—H18B	0.9600
S1—C8	1.816 (2)	C18—H18C	0.9600
O3—C12	1.385 (3)	C19—C22	1.529 (4)
O3—H3	0.806 (18)	C19—C21	1.534 (4)
N1—C26	1.474 (4)	C19—C20	1.538 (4)
N1—C27	1.476 (4)	C20—H20A	0.9600
N1—H1A	0.890 (17)	C20—H20B	0.9600
N1—H1B	0.892 (17)	C20—H20C	0.9600
C2—C3	1.387 (3)	C21—H21A	0.9600
C2—C7	1.409 (3)	C21—H21B	0.9600
C3—C4	1.364 (4)	C21—H21C	0.9600
C3—H3A	0.9300	C22—H22A	0.9600
C4—C5	1.366 (4)	C22—H22B	0.9600
C4—H4	0.9300	C22—H22C	0.9600
C5—C6	1.383 (3)	C23—C24	1.450 (5)
C5—H5	0.9300	C23—H23A	0.9600
C6—C7	1.389 (3)	C23—H23B	0.9600
C6—H6	0.9300	C23—H23C	0.9600
C8—C9	1.504 (3)	C24—C25	1.513 (5)
C8—H8A	0.9700	C24—H24A	0.9700
C8—H8B	0.9700	C24—H24B	0.9700
C9—C10	1.374 (3)	C25—C26	1.499 (4)
C9—C14	1.379 (3)	C25—H25A	0.9700
C10—C11	1.393 (3)	C25—H25B	0.9700
C10—H10	0.9300	C26—H26A	0.9700
C11—C12	1.400 (3)	C26—H26B	0.9700
C11—C19	1.543 (3)	C27—C28	1.521 (4)
C12—C13	1.405 (3)	C27—H27A	0.9700
C13—C14	1.392 (3)	C27—H27B	0.9700
C13—C15	1.538 (4)	C28—C29	1.490 (5)
C14—H14	0.9300	C28—H28A	0.9700
C15—C17	1.526 (5)	C28—H28B	0.9700
C15—C18	1.532 (4)	C29—C30	1.498 (5)
C15—C16	1.538 (4)	C29—H29A	0.9700
C16—H16A	0.9600	C29—H29B	0.9700
C16—H16B	0.9600	C30—H30A	0.9600
C16—H16C	0.9600	C30—H30B	0.9600
C17—H17A	0.9600	C30—H30C	0.9600
			
O2—C1—O1	122.1 (3)	H18A—C18—H18C	109.5
O2—C1—C2	119.5 (2)	H18B—C18—H18C	109.5
O1—C1—C2	118.4 (2)	C22—C19—C21	110.4 (2)
C7—S1—C8	103.55 (11)	C22—C19—C20	107.2 (2)
C12—O3—H3	114 (3)	C21—C19—C20	106.7 (2)
C26—N1—C27	114.0 (2)	C22—C19—C11	111.4 (2)
C26—N1—H1A	109.4 (18)	C21—C19—C11	109.5 (2)
C27—N1—H1A	108.8 (18)	C20—C19—C11	111.5 (2)
C26—N1—H1B	107.3 (18)	C19—C20—H20A	109.5
C27—N1—H1B	106.9 (18)	C19—C20—H20B	109.5
H1A—N1—H1B	110 (3)	H20A—C20—H20B	109.5
C3—C2—C7	118.5 (2)	C19—C20—H20C	109.5
C3—C2—C1	118.1 (2)	H20A—C20—H20C	109.5
C7—C2—C1	123.4 (2)	H20B—C20—H20C	109.5
C4—C3—C2	122.5 (2)	C19—C21—H21A	109.5
C4—C3—H3A	118.8	C19—C21—H21B	109.5
C2—C3—H3A	118.8	H21A—C21—H21B	109.5
C3—C4—C5	119.2 (2)	C19—C21—H21C	109.5
C3—C4—H4	120.4	H21A—C21—H21C	109.5
C5—C4—H4	120.4	H21B—C21—H21C	109.5
C4—C5—C6	120.2 (3)	C19—C22—H22A	109.5
C4—C5—H5	119.9	C19—C22—H22B	109.5
C6—C5—H5	119.9	H22A—C22—H22B	109.5
C5—C6—C7	121.3 (2)	C19—C22—H22C	109.5
C5—C6—H6	119.4	H22A—C22—H22C	109.5
C7—C6—H6	119.4	H22B—C22—H22C	109.5
C6—C7—C2	118.3 (2)	C24—C23—H23A	109.5
C6—C7—S1	120.62 (18)	C24—C23—H23B	109.5
C2—C7—S1	121.05 (17)	H23A—C23—H23B	109.5
C9—C8—S1	107.30 (17)	C24—C23—H23C	109.5
C9—C8—H8A	110.3	H23A—C23—H23C	109.5
S1—C8—H8A	110.3	H23B—C23—H23C	109.5
C9—C8—H8B	110.3	C23—C24—C25	116.3 (4)
S1—C8—H8B	110.3	C23—C24—H24A	108.2
H8A—C8—H8B	108.5	C25—C24—H24A	108.2
C10—C9—C14	118.3 (2)	C23—C24—H24B	108.2
C10—C9—C8	121.6 (2)	C25—C24—H24B	108.2
C14—C9—C8	120.1 (2)	H24A—C24—H24B	107.4
C9—C10—C11	122.7 (2)	C26—C25—C24	112.1 (3)
C9—C10—H10	118.6	C26—C25—H25A	109.2
C11—C10—H10	118.6	C24—C25—H25A	109.2
C10—C11—C12	117.1 (2)	C26—C25—H25B	109.2
C10—C11—C19	120.4 (2)	C24—C25—H25B	109.2
C12—C11—C19	122.5 (2)	H25A—C25—H25B	107.9
O3—C12—C11	118.8 (2)	N1—C26—C25	112.2 (2)
O3—C12—C13	118.9 (2)	N1—C26—H26A	109.2
C11—C12—C13	122.2 (2)	C25—C26—H26A	109.2
C14—C13—C12	117.0 (2)	N1—C26—H26B	109.2
C14—C13—C15	120.0 (2)	C25—C26—H26B	109.2
C12—C13—C15	122.9 (2)	H26A—C26—H26B	107.9
C9—C14—C13	122.7 (2)	N1—C27—C28	111.4 (2)
C9—C14—H14	118.7	N1—C27—H27A	109.3
C13—C14—H14	118.7	C28—C27—H27A	109.3
C17—C15—C18	110.2 (3)	N1—C27—H27B	109.3
C17—C15—C13	109.0 (3)	C28—C27—H27B	109.3
C18—C15—C13	112.2 (2)	H27A—C27—H27B	108.0
C17—C15—C16	107.4 (3)	C29—C28—C27	113.0 (3)
C18—C15—C16	106.9 (3)	C29—C28—H28A	109.0
C13—C15—C16	111.0 (2)	C27—C28—H28A	109.0
C15—C16—H16A	109.5	C29—C28—H28B	109.0
C15—C16—H16B	109.5	C27—C28—H28B	109.0
H16A—C16—H16B	109.5	H28A—C28—H28B	107.8
C15—C16—H16C	109.5	C28—C29—C30	113.4 (3)
H16A—C16—H16C	109.5	C28—C29—H29A	108.9
H16B—C16—H16C	109.5	C30—C29—H29A	108.9
C15—C17—H17A	109.5	C28—C29—H29B	108.9
C15—C17—H17B	109.5	C30—C29—H29B	108.9
H17A—C17—H17B	109.5	H29A—C29—H29B	107.7
C15—C17—H17C	109.5	C29—C30—H30A	109.5
H17A—C17—H17C	109.5	C29—C30—H30B	109.5
H17B—C17—H17C	109.5	H30A—C30—H30B	109.5
C15—C18—H18A	109.5	C29—C30—H30C	109.5
C15—C18—H18B	109.5	H30A—C30—H30C	109.5
H18A—C18—H18B	109.5	H30B—C30—H30C	109.5
C15—C18—H18C	109.5		

### Hydrogen-bond geometry (Å, °)

**Table:**

*D*—H···*A*	*D*—H	H···*A*	*D*···*A*	*D*—H···*A*
O3—H3···O1^i^	0.81 (2)	2.02 (2)	2.723 (3)	146 (3)
N1—H1A···O2	0.89 (2)	1.88 (2)	2.767 (3)	177 (3)
N1—H1B···O1^ii^	0.89 (2)	2.00 (2)	2.806 (3)	150 (3)
N1—H1B···O2^ii^	0.89 (2)	2.36 (2)	3.167 (3)	150 (2)
N1—H1B···O2^ii^	0.89 (2)	2.36 (2)	3.167 (3)	150 (2)

Symmetry codes: (i) *x*+1, *y*, *z*; (ii) −*x*+1, −*y*, −*z*+1.
